# Giant tonsillolith causing odynophagia in a child: a rare case report

**DOI:** 10.1186/1757-1626-1-50

**Published:** 2008-07-18

**Authors:** Jagdeep S Thakur, Ravinder S Minhas, Anamika Thakur, Dev R Sharma, Narinder K Mohindroo

**Affiliations:** 1 Department of Otolaryngology-Head Neck Surgery, I. G. Medical College, Shimla, HP, 171001, India; 2 Dept of Pharmacology, I G Medical College, Shimla, HP, 171001, India

## Abstract

Giant tonsillolith is a rare clinical entity. Commonly, it occurs between 20–77 years of age. We had a twelve years old female patient, who had odynophagia due to a giant tonsillolith. The stone was removed and tonsillectomy was performed. We reviewed the literature on this rare clinical entity and found that this is the fourth case of giant tonsillolith in a child and largest ever tonsillolith to be reported in English literature.

## Background

Giant tonsillolith is a rare entity [1] although small concretions in the palatine tonsil are a common clinical finding in adults [2]. These patients usually present with bad breath odor, pain during swallowing or foreign body sensation in the throat. In English literature, there are many reports on tonsillolith in adults but we found ('Medline' and 'Scopus' search) only three reports on tonsillolith in children [3-5]. We report a case of giant tonsillolith and review the literature on this rare clinical finding.

## Case presentation

A 12 years female child presented with pain during swallowing for last one year. The pain was mild-moderate and non-radiating. Patient had recurrent episodes of sore throat for last six years and used to recover after medication. There were no other associated symptoms. The medical and family histories were insignificant.

On examination, both tonsils were enlarged but with massive enlargement of left tonsil. There were no other significant findings on examination of oral cavity, oropharynx or larynx. The provisional diagnosis of recurrent tonsillitis was made and patient was advised tonsillectomy under general anesthesia.

**Figure 1:**
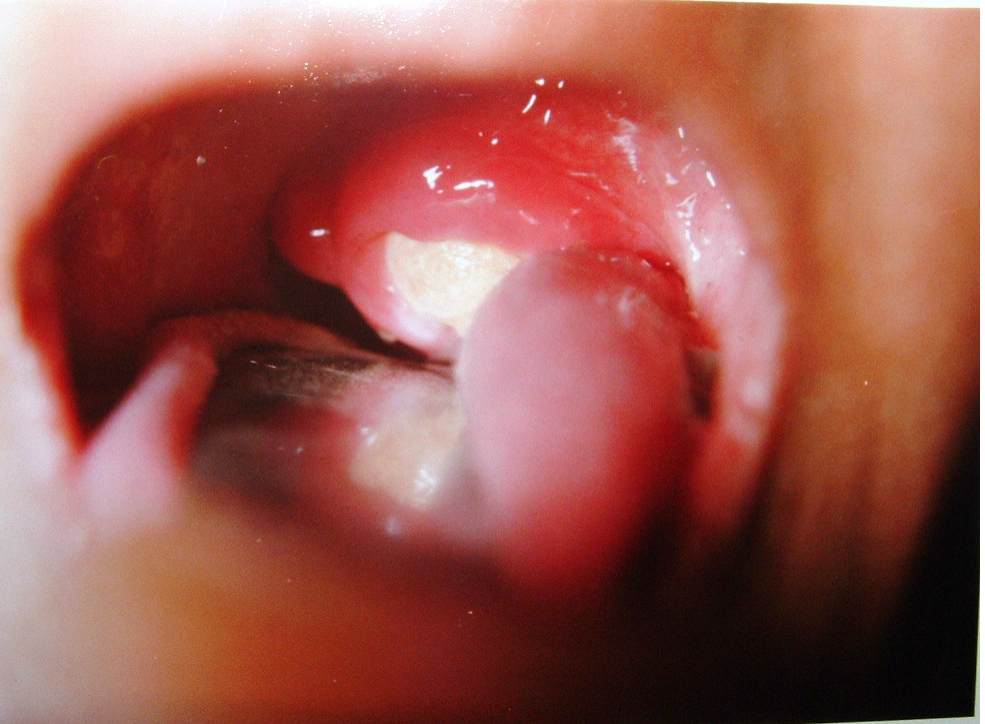
Large stone visible on intra oral examination.

**Figure 2:**
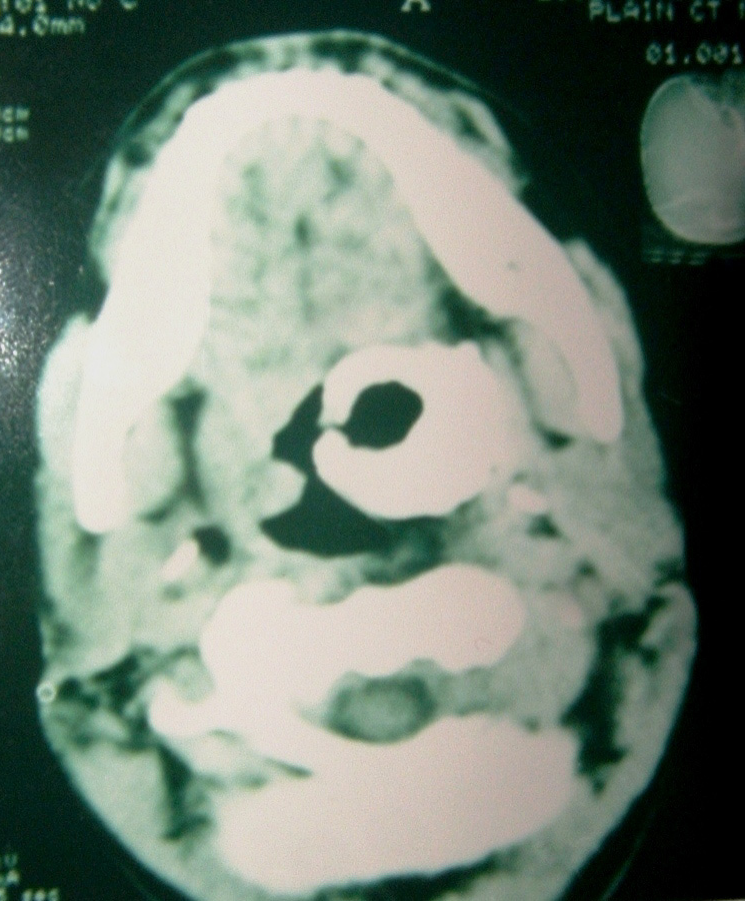
CT scan showing stone in oral cavity.

**Figure 3:**
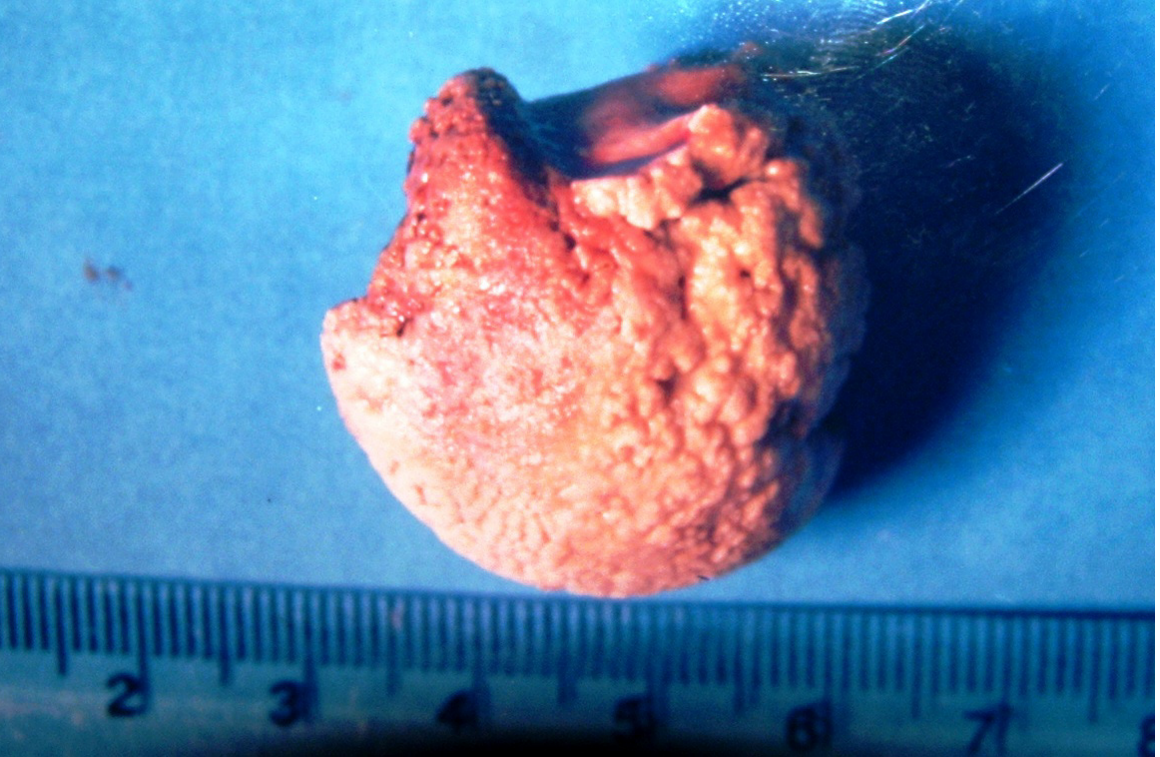
Photograph of removed stone.

After three weeks at the time of admission, patient had large chalky white patch on the left tonsil which was hard and could not be removed (Fig 1). The tonsil was tender on touch. Patient underwent radiological examination and X-ray and CT scan (Fig 2) showed large 3.9 × 3.4 cm radio opaque shadow in left tonsillar area. This mass had no relation to styloid process or any bone. The provisional diagnosis of giant tonsillolith was made and surgical removal of stone with tonsillectomy was done under general anesthesia. The stone (Fig 3) was hard, yellowish-white with 4.2 × 3.6 × 2.1 cm in size and was made of calcium carbonate and oxalate. Postoperative period was uneventful and patient recovered well. Patient had complete relief in odynophagia with follow up lasting for one year.

## Discussion

The tonsilloliths are white or yellow colored stones composed of calcium salts such as hydroxyapatite or calcium carbonate apatite, oxalates and other magnesium salts or containing ammonium radicals [6]. The exact pathogenesis of these stones is unknown although there are many hypotheses on the formation of these calculi. It has been stated that they originate as a result repeated tonsillitis which lead to fibrosis of ducts of crypts and retention of epithelial debris thereof. This epithelial debris forms the ideal media for the growth of bacterial, actinomyces [7] and fungi such as *Leptothrix buccalis *[8]. Finally dystrophic calcification occurs as a result of deposition of above stated inorganic salts from the saliva secreted in the mouth by major and minor salivary glands.

Calculi have been reported in the peritonsillar region [9] and lateral pharyngeal wall [10]; and were explained by calcification of peritonsillar abscess, presence of ectopic tonsillar tissue and calcification of saliva in blocked secretory ducts of minor salivary glands [11,12].

We did 'Medline' and 'Scopus' search with keywords: 'Tonsillectomy'; Tonsillolith'; 'Child'; 'Pediatrics' and found only three reports [3-5] on tonsillolith in pediatric age group. Tonsilloliths are rare in pediatric age group as they occur between 20 and 77 years of age [10,13-15]. The stone may be asymptomatic or can cause variety of symptoms i.e. bad breath odor, foreign body sensation in throat, odynophagia or dysphagia. These stones are usually found in X-ray or CT scan done for other reason [15]. X ray shows single or multiple radio opaque shadows which can be mistaken for foreign body, calcified lymph node, unerupted tooth, calcified stylohyoid ligament or prominent tuber of maxilla or elongated styloid process [15]. Computed tomography (CT) is found to diagnostic by obtaining multiple axial sections [2]. We also found the stone by chance as calculus was extruding out from tonsil and it was only CT scan which confirmed the diagnosis.

It has been advocated to remove the stone surgically or perform tonsillectomy if stone is large or impacted within tonsil [8]. In our case although stone was quite large and was removed as whole but tonsillectomy was also performed as tonsils were hypertrophied.

## Conclusion

In this case study and review of literature we concluded that:

1. The hypothesis of recurrent tonsillitis leading to fibrosis of ducts and formation of tonsillolith appeared to be reason for the tonsillolith in our case as patient had recurrent tonsillitis for last six years.

2. All the pediatric or adult patients presenting with foreign body sensation in throat, dysphagia or odynophagia should have thorough tonsillar examination including digital palpation to rule out any concretion or stone in tonsil as a cause of above mentioned symptoms and CT scan should be done to confirm this rare diagnosis.

## Consent

The written informed consent of the guardian of the patient has been obtained for the publication of this case report and accompanied images.

## Competing interests

The authors declare that they have no competing interest.

## Authors' contributions

JST has designed and written the article and is the principal contributor, RSM was involved with the management of the patient, conception, design and review of the article, AT was involved in acquisition of the data, review of the literature and critical review of the article, DRS was involved in the management of the patient, conception and critical review of the article, NKM was also involved in the management of the patient, conception and critical review of the article. All the authors have read and given final approval for this article.
